# Prevalence of Frank’s sign in healthy young individuals, morphological characteristics, and its association with family history of chronic disease

**DOI:** 10.1007/s12024-024-00868-7

**Published:** 2024-08-02

**Authors:** Feyza Aksu, Ramazan Fazil Akkoc

**Affiliations:** Department of Anatomy, Faculty of Medicine, Firat University, Elazig, Turkey

**Keywords:** Frank’s sign (FS), Earlobe crease (ELC), Preventive medicine, Morphology

## Abstract

Frank’s sign (FS, earlobe crease) is known to be associated with many chronic diseases i.e. coronary, cerebrovascular, and peripheral vascular diseases. The objective of this study is to investigate the presence and grade of FS in healthy, young-adult individuals aging between 18 and 25. Furthermore, to evaluate the relationship between FS and the chronic disease history of first-degree family members. 853 healthy people with a mean age of 20.09 years were imvolved in the research on a voluntary basis. Of 853 individuals, 85.3% (n:728) had no FS, whereas 14.7% (n:125) had. Of the 125 people observed FS, 41.6% (n:52) were female and 58.4% (n:73) were male. Of the 125 people diagnosed with FS, 32.8% (n:41) had no family history of any chronic disease, and 67.2% (n:84) had at least 1 family history of chronic disease as well. It was determined that there was a statistically significant (*p* < 0.05) relationship between FS identified in healthy individuals and familial chronic disease history. In this study, while no FS was observed in 85.3% of the participants, grade 1 was detected in 12.4% (n:105), grade 2a in 1.9% (n:16), grade 2b in 0.2% (n:2), and grade 3 in 0.2% (n:2). To the best of our knowledge, this study investigated the relationship between the presence and grade of FS in the healthy, young, Turkish population and the presence/absence of chronic disease in the first-degree relatives of individuals for the very first time. As a result of larger studies, being aware of the presence of FS, especially in young healthy individuals, may help predict some chronic diseases, and caution may be taken to avoid these diseases at an early age in at-risk individuals.

**Supplementary Information:**

The online version contains supplementary material available at 10.1007/s12024-024-00868-7.

## Introduction

Despite Frank’s sign (FS, earlobe crease), drew attention since the busts of the Roman Emperor Hadrian, who was known to have heart condition in history, it was first identified by Sanders T. Frank in 1973 [1]. It was suggested that FS is a substantial predictor of various diseases i.e. coronary, cerebrovascular, and peripheral vascular diseases [2].

FS is not congenital. Age-related alterations occur in the earlobe of humans. Since age-related alterations and wrinkles in older ages may appear like FS even without cardiovascular disease, FS is considered to be more practical in terms of diagnosis, especially in people under the age of 60 [3–5].

Many theories regarding the pathophysiology of FS have been argued in the literature. In 1980s, it was reported that FS could result from insufficient arterial supply as localized on the earlobe [6]. In 1990s, it was stated that there was an association between macrophage activity, which plays a role in atherosclerosis, ageing, and the preservation of earlobe collagen. Moreover, FS and cardiovascular diseases have been reported to be associated with elastin loss and tearing [7]. In genetic studies on FS, it has been reported that it is related to HLA-B27 and C3-F genes and chromosome 11 [8]. On the other hand, FS has been associated with the presence of shortened telomeres, which indicate ageing, in peripheral white blood cells [9]. In another study it has been reported that free radicals elevated in circulation due to oxidative stress cause elastic fibre loss, increased blood vessel intima-media thickness, and decreased blood flow in the earlobe without collateral circulation. Similar pathological changes have been reported to monitor in weakened coronary arteries [10].

In a study examining the histology of FS, it has been reported that a significant correlation existed between the heart and morphological modifications in the earlobe. Embryologically, the earlobe is formed by the integration of the first and sixth auricular hillocks on the first and second pharyngeal arches. This formation makes the earlobe susceptible to chronic hypoxia and reoxygenation damage before the acute event and signs of cyanosis appeared in the distal part of the body in epidermal-dermal degeneration. Solar elastosis, myoelastofibrosis, and degeneration similar to Wallerian degeneration in peripheral nerves have been stated in FS histological examination [5].

FS grading is described according to the crease’s moving forward, starting from the tragus and around the earlobe, and depth of crease. Grade 1 is a mild crease in the skin less than 1/3 of the way from the tragus to the earlobe. Grade 2a is a superficial skin crease extending at least halfway from the tragus to the earlobe. Grade 2b is a superficial skin crease that completely covers the area between the tragus and the earlobe. Grade 3 is a deep skin crease covering the entire earlobe [11].

In one research, a 52-year-old male patient, who was a non-smoker and had no cardiac history or complaints and was planned to undergo laparoscopic radical prostatectomy, was presented. It was stated that 1 h after the beginning of the surgery, tachycardia and new pathological findings were detected on the ECG. The patient, who had bilateral FS, subsequently underwent elective coronary stent placement. On the basis of these data, it was reported that FS might be a practical marker for coronary artery disease in cases where the patient’s history could not be taken adequately and accurately in the pre-anesthesia assessment [12].

In previous studies, it has been stated that FS is associated with diabetes mellitus (DM), hypertension (HT), myocardial infarction (MI), and coronary artery disease (CAD) in both males and females. Additionally, in terms of these diseases, it has been stated that individuals with FS yet who are asymptomatic are at high risk [12–17].

To the best of our knowledge, there has been no study in the literature investigating the presence and grade of FS in healthy young adults aging 18–25 in the Turkish population. The objective of this study was to evaluate the presence and grade of FS in young adult individuals aging 18–25 who do not have a chronic disease (to eliminate alterations caused by advanced age). Moreover, the relationship between the presence of FS and the presence of chronic diseases in the family members (mother, father, and siblings) was examined.

## Materials and methods

In this cross-sectional study, healthy young individuals aged 18–25 years who were continuing their education at Fırat University were included voluntarily. The age and gender of healthy individuals, and whether their first-degree family members (mother, father, and siblings) had chronic diseases were interrogated. The presence of Frank’s Sign in both ears of volunteer individuals was assessed independently by two different observers. Individuals with any chronic disease, earlobe pathology, and consecutive earring holes in the earlobe were excluded from the research. The classification of FS according to grades was based on the study by Patel et al. [11].

### Statistical analysis

IBM SPSS Statistics Version 22.0 package program was utilized in the statistical analysis of the data. Descriptive statistics were presented as mean ± standard deviation. Pearson Chi-square test was utilized to compare categorical variables between groups, and the Mann-Whitney U test was used to compare two independent groups in quantitative measurements. In all tests, the statistical significance level was considered to be 0.05.

## Results

A total of 853 people, 419 (49.1%) males and 434 (50.9%) females, were included in the research on a voluntary basis. The mean age of the participants was 20.09 ± 1.67 and all were healthy. The number of chronic diseases that the family members (mothers, fathers, and siblings) of healthy participants had was questioned. The number of people without a chronic disease in their family was 567 (66.5%), the number of people with 1 chronic disease was 202 (23.7%), the number of people with 2 chronic diseases was 69 (8.1%), the number of people with 3 chronic diseases was 13 (1.5%), and the number of people with 4 chronic diseases was 2 (0.2%). FS was absent in 728 (85.3%) of 853 people, whereas it was present in 125 (14.7%).

Of the 125 people with FS, 52 (41.6%) were female and 73 (58.4%) were male. The prevalence of FS was noted to be higher in males (males: 17.4%; females: 12.0%), whereas a statistically significant difference was found with regard to gender (*p* = 0.016) (Table 1).

**Table 1:** Data about the participants

		Mean age (year)	Female (*n*)	Male (*n*)	No history of any chronic disease in the family (*n*)	Having at least one chronic disease history in the family (*n*)
Frank’s sign	No	20.04	382	346	526	202
	Yes	20.39	52	73	41	84

The mean age of the participants diagnosed with FS (20.39 ± 1.67) was detected to be higher than those without FS (20.04 ± 1.65), and this outcome was statistically significant (*p* < 0.05) (Table 1).

While 41 (32.8%) of the 125 people with FS did not have a family history of any chronic disease, 84 (67.2%) had a family history of at least 1 chronic disease. As a result of these data, it was determined that there was a statistically significant (*p* < 0.05) relationship between FS identified in healthy individuals and familial chronic disease history. While 526 (72.3%) of the 728 people without FS did not have a family history of any chronic disease, 202 (27.7%) had a family history of at least 1 chronic disease (Table 1).

**Fig. 1:**
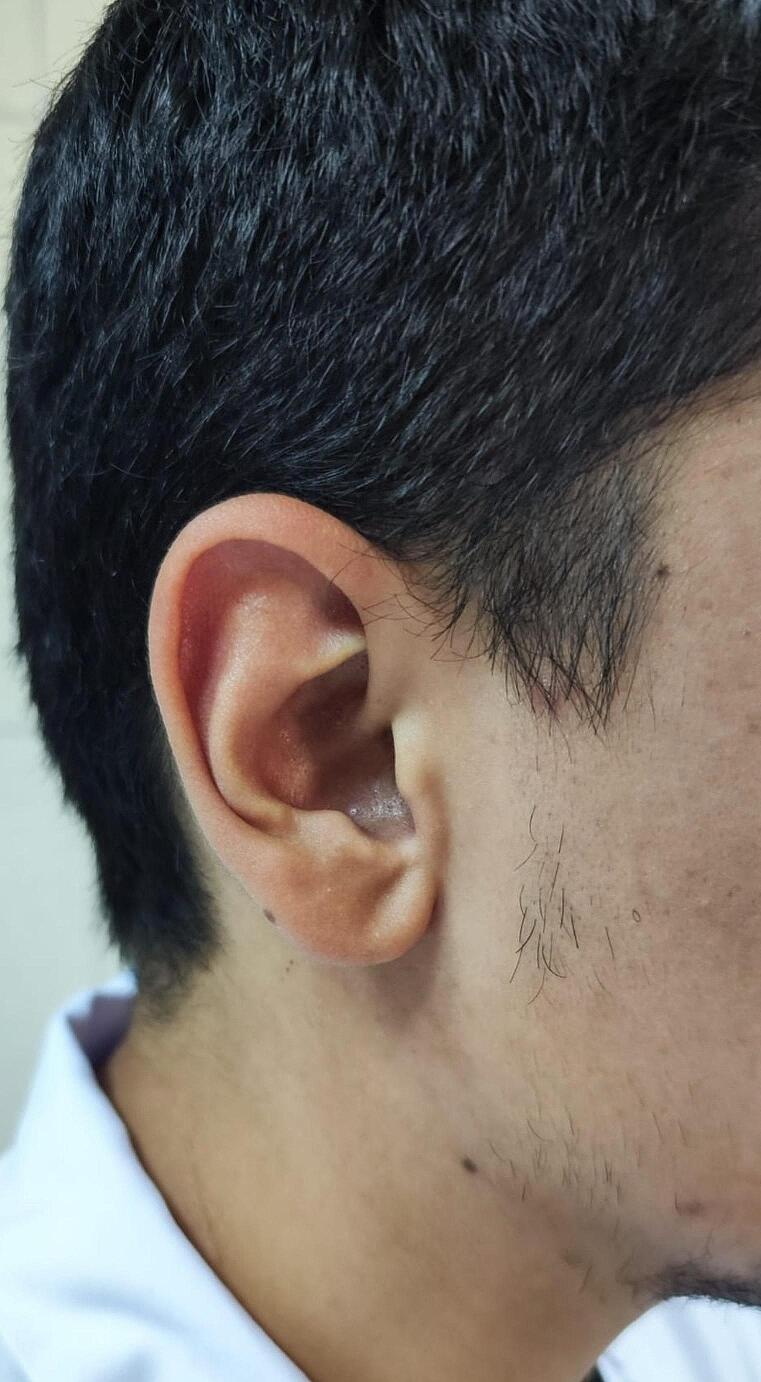
Right-sided Frank’s sign (grade 2b)

FS existed in 125 of the healthy participants included in the study. Considering FS grading, grade 1 was the most prevalent with a rate of 84%. 122 people had bilateral FS. In the remaining 3 individuals, it was unilateral and in the right ear, and all of them were in the grade 1 group. The distribution of FS grades by gender was presented in Table 2 (Fig. 1).

**Table 2:** Distribution of FS grades by gender

	Grade 1	Grade 2a	Grade 2b	Grade 3	Total
Female	46 (% 43.8)	5 (% 31.3)	1 (% 50)	0 (% 0)	52 (% 41.6)
Male	59 (% 56.2)	11 (% 68.7)	1 (% 50)	2 (% 100)	73 (% 58.4)
Total	105 (% 100)	16 (% 100)	2 (% 100)	2 (% 100)	125 (% 100)

## Discussion

FS, which was also remarked in historical busts, was first defined by Sanders T. Frank in 1973 [1]. Since it has been defined, many studies have been conducted to explain its relationship with various diseases [13–17].

While FS is not congenital, its prevalence increases in some chronic diseases and with aging. In this research, the presence of FS in healthy and young (18–25 years old) adults was examined for the very first time in the Turkish population, to the best of our knowledge. Furthermore, the presence of a chronic disease in the first-degree relatives of the people whose FS was investigated was questioned and its association with FS was assessed.

Reviewing most of the studies on FS in the literature only in terms of sample age range, the mean age was generally around 60 years old or older [18–20]. In an autopsy study including 520 people, one of the groups examined people under the age of 40; however, it should be noted that FS is not detected in newborns and becomes visible with advanced age [3, 21]. In this research, the average age of the sampling was 20, and the pediatric period when FS does not exist or is quite rare, and the older age periods when it is very prevalent, or the normal anatomical structure is disrupted were not included. In this regard, it is distinguished from the literature.

In previous studies examining the presence of FS, the majority of the samples consisted of individuals with chronic diseases, especially individuals with coronary artery disease or control group individuals with a history of chronic diseases (excluding cardiac diseases) or individuals who died from different reasons regardless of disease [13, 14, 18–20]. In this study, on the other hand, none of the participants had any chronic disease and all were healthy individuals.

The earlobes of children and young adults are normally smooth. The prevalence of FS increases with aging [5]. Nonetheless, some studies have demonstrated that FS is substantial in indicating atherosclerosis, regardless of age [14, 22]. The prevalence of FS varies by society. In a systematic review of 13 cross-sectional studies involving various ethnic populations, mostly North American and Chinese populations, the prevalence of FS was reported to be 60.5%. This systematic review reported that the prevalence of FS was the lowest with a rate of 17% in the Japanese population and the highest in the North American population with a rate of 73% [23]. In an autopsy study including 520 individuals, the prevalence of FS was reported to be 55.0%. In the same study, this rate was close to 20% in individuals under the age of 40, whereas it was around 75% in individuals over the age of 60 [23]. In this study, on the other hand, the of FS was noted to be 14.7%. This ratio is slightly below the prevalence of FS in the literature. We believe that this is due to the fact that the individuals creating the sampling group are young adults and healthy individuals between the ages of 18–25.

In this study population, the female gender ratio (50.9%) was higher than that of males. Nevertheless, examining the distribution of FS detected by gender, it was noted to be more frequent in males with a rate of 58.4%. In most of the studies in the literature, as in this study, it has been reported that the FS rate identified is higher in males, despite it varies in different percentages (between 55 and 75%) [13, 20, 24, 25]. However, an autopsy study reported that the FS ratio was higher in females [21].

In terms of FS grading, in a study carried out on 165 individuals in the literature, it was reported that FS did not exist in 27 (16.4%) people, grade 1 was reported in 12 (7.3%) people, grade 2a was reported in 18 (10.9%) people, grade 2b was reported in 53 (32.1%) people, and grade 3 was reported in 55 (33.3%) people. This study reported on 165 deceased individuals with a mean age of 61. In the same study, the FS grading of 50 people whose average age was 63 years and above and whose cause of death was sudden cardiac death or death related to a cardiac cause was also indicated. Of these, 11 (22%) were reported to be grade 0–1 or 2a, and 39 (78%) were reported to be grade 2b or 3 [26]. In this study conducted on 853 individuals, while FS was not present in 728 (85.3%) of the participants, it was noted as grade 1 in 105 (12.4%), grade 2a in 16 (1.9%), grade 2b in 2 (0.2%), and grade 3 in 2 (0.2%). This study data and literature data differ from each other. This is because even though the sampling was large in previous studies, the mean age was mostly 60 years and older, and accompanying chronic diseases were reported; in this study, the mean age was approximately 20 (18–25) and all participants were healthy individuals.

In light of these data, the presence of FS and its advanced grade (grade 2b or 3) is probable in older people who are diagnosed with the disease or are symptomatic, and its prevalence is high. In this study, it was revealed that FS may also be observed in young individuals without any disease; however, its grade is generally low (grade 1 or 2a), indicating that preventive medicine should not be ignored in the healthy young generation.

Gasga and Phan [27], reported FS in a 66-year-old male patient with a history of MI who applied with chest pain complaint. In the meantime, it was reported that the patient’s father also had a history of MI and the presence of FS [27]. In another research, 41 male patients with a history of MI (59% had FS, 41% had no FS) and 134 first-degree relatives of the patients were examined. All relatives of 41 patients with MI who were diagnosed with cardiac pathology were reported to have FS, approximately half of parents without clinical cardiac pathology had FS, and approximately one-fourth of healthy siblings had FS [28]. In this study, 41 (32.8%) of the 125 people diagnosed with FS did not have a history of any chronic disease in their family members, whereas 84 (67.2%) had a history of at least 1 chronic disease in their family members. In the literature, family history has been investigated in elderly individuals with a history of chronic diseases with FS. The difference between this study and the literature is that whether healthy young individuals have FS, we assessed its relationship with family history, and this relationship was found to be significant.

## Conclusion

In this research, the presence and grade of FS were examined for the first time in a healthy young population. Furthermore, the relationship between the presence of FS and the presence/absence of chronic disease in the first-degree relatives of the individuals were investigated. As a result of larger studies, being aware the presence of FS, particularly in young healthy individuals, may help predict some chronic diseases i.e. DM, HT, MI and CAD. Moreover, precautions may be taken to avoid the disease at a young age in people at risk.

### Key points

1. In this study, the presence of FS in healthy and young adults (18–25 years old) was investigated for the first time in the Turkish society.
2. The prevalence of FS in healthy young adults was 14.7%.
3. 32.8% of healthy young people with FS did not have a family history of any chronic disease.
4. 67.2% of healthy young people with FS had a family history of at least 1 chronic disease.

## Electronic supplementary material

Below is the link to the electronic supplementary material.

Supplementary Material 1
