# Supplementary material for: Prevalence of Frank’s sign in healthy young individuals, morphological characteristics, and its association with family history of chronic disease
Source: Forensic Sci Med Pathol. 2024 Aug 2;20(4):1187–92. doi: 10.1007/s12024-024-00868-7 (PMC11790723; doi:10.1007/s12024-024-00868-7)
Supplement: Supplementary file 1 — Supplementary Material 1 [file 12024_2024_868_MOESM1_ESM.pdf]

protranslate

American Translators Association
MEMBER : #279427

Date: **20.06.2024**

Certification of Translation Accuracy

Document Type:	**Prevalence of Frank's Sign in Healthy Young Individuals, Morphological Characteristics, and Its Association with Family History of Chronic Disease**		
Translation Date:	**20.06.2024**	Order:	**#357426**
Source Language:	**Turkish**	Target Language	**English**

We, Protranslate, a professional online translation agency, hereby certify that the above-mentioned document has been translated by an experienced, certified, and competent professional translator and that, to the best of our knowledge, the translated document reflects the content, meaning, and style of the original document and is an accurate and complete translation of the original document.

This is to certify the accuracy of the translation only. We do not make any claims regarding the authenticity of the original document or its contents. In compliance with our Terms and Conditions, Protranslate assumes no liability for the manner in which the translation is used by the client or any other party.

A copy of the translation is attached herewith.

Regards,

PROTRANSLATE CERTIFIED

ISO 17100:2015
CERTIFIED TRANSLATION AND INTERPRETATION SERVICES
Certificate No : C-0005
Certification Intial Issue Date : 20.02.2023
Certification Issue Date : 19.02.2024
Certificate Validitation Date : 19.02.2025
Rev Date : --
Rev. No : 00
Approved

100% CERTIFIED 100% HUMAN QUALITY GUARANTEE

Protranslate Translation Services
